# Impact of Copper Oxide Nanoparticles on Adventitious Shoot Regeneration, Axillary Shoot Multiplication, Rooting, and Bioactive Compounds in *Ajuga multiflora* Bunge

**DOI:** 10.3390/plants14243807

**Published:** 2025-12-13

**Authors:** Iyyakkannu Sivanesan, Shuchi Upadhyay, Young-Soo Keum, Se Chul Chun, Ramesh Kumar Saini

**Affiliations:** 1 Department of Environmental Health Science, Human and Eco Care Center, Konkuk University, 1, Hwayang-dong, Gwangjin-gu, Seoul 05029, Republic of Korea; siva74@konkuk.ac.kr (I.S.); scchun@konkuk.ac.kr (S.C.C.); 2 School of Health Sciences and Technology, UPES, Dehradun 248007, Uttarakhand, India; shuchi.upadhyay@ddn.upes.ac.in; 3 Department of Crop Science, Konkuk University, 1, Hwayang-dong, Gwangjin-gu, Seoul 05029, Republic of Korea; rational@konkuk.ac.kr

**Keywords:** in vitro propagation, metal oxide nanoparticles, carotenoids, 22-dehydroclerosterol, clerosterol, fatty acids, tocopherol

## Abstract

The present study investigated the effects of copper oxide nanoparticles (CuO NPs) at concentrations of 0, 5, 10, 20, and 40 mg/L on micropropagation and the accumulation of lipophilic metabolites in *Ajuga multiflora*, a medicinally valuable ornamental species. The highest number of adventitious shoots (29.4 shoots per explant) was obtained on the shoot induction medium with 5 mg/L CuO NPs. Shoot production gradually decreased at higher CuO NPs concentrations, falling to just 1.1 shoots per explant at 40 mg/L CuO NPs. A similar pattern was seen in axillary shoot multiplication (22.4 shoots per explant at 5 mg/L CuO NPs). However, the maximum shoot fresh weight (0.269 g) was reached on the shoot multiplication medium containing 10 mg/L CuO NPs. Root induction was most effective at 5–10 mg/L CuO NPs, while higher concentrations (20 or 40 mg/L CuO NPs) suppressed or inhibited root formation and altered plantlet morphology. Notably, this study is among the first to assess CuO NPs’ effects across multiple regeneration stages rather than focusing on just one morphogenic event. This emphasizes the importance of optimizing the dose not only for initial shoot induction but also for later multiplication and rooting, ensuring effective micropropagation. Metabolite analysis showed that both the type of organ (microshoots vs. leaves) and CuO NPs concentration significantly affected the levels of α-tocopherol, carotenoids, sterols, and fatty acids. Leaves had higher amounts of α-tocopherol and total carotenoids compared to microshoots. The phytosterol levels also varied, with leaves containing more 22-dehydroclerosterol and total phytosterols, while microshoots had more clerosterol. Treatment with 5 mg/L CuO NPs increased phytosterol accumulation in both organs. CuO NPs significantly influenced the fatty acid profiles. In microshoots, total polyunsaturated fatty acids (PUFAs) increased and total saturated fatty acids (SFAs) decreased with higher CuO NPs levels. Conversely, in leaves, higher CuO NPs concentrations led to increased SFAs and decreased PUFAs, along with a significant rise in the omega-6 (n-6)/n-3 PUFAs ratio. These findings suggest that controlled application of CuO NPs can serve as an elicitor to boost phytochemical production during micropropagation.

## 1. Introduction

Nanotechnology provides innovative uses in horticulture and plant biotechnology [1,2]. The use of nanoparticles (NPs) boosts plant growth, yield, and quality, as well as seed germination and stress tolerance, by enhancing nutrient uptake, scavenging reactive oxygen species, modifying biochemical content, maintaining hormonal balance, changing enzyme activity, and influencing gene expression [1]. Furthermore, NPs can improve the success of different stages of micropropagation, secondary metabolite production, and genetic transformation [2,3,4,5].

The interaction between NPs and plants triggers a range of physiological and biochemical responses that can promote or inhibit growth, depending on NP composition, size, concentration, and agglomeration [6]. Upon entering plant cells, tissues, and organs, NPs influence nutrient uptake, photosynthetic efficiency, and water balance by altering ion transport and membrane permeability [7,8]. Biochemically, exposure to nanoparticles can disrupt cellular redox balance, often resulting in the production of reactive oxygen species. In turn, these reactive oxygen species activate antioxidant defense mechanisms, including enzymes like catalase, peroxidases, and superoxide dismutase, as well as non-enzymatic compounds like ascorbate, carotenoids, flavonoids, glutathione, phenolics, and tocopherols [8,9]. These interactions also affect metabolic pathways, phytohormone signaling networks, including abscisic acid, auxin, cytokinin, ethylene, and gibberellin, and the synthesis of secondary metabolites like alkaloids, anthocyanins, flavonoids, phenolics, and terpenoids. Ultimately, these changes influence processes such as morphogenesis (cell division, elongation, and differentiation), pigment production (e.g., chlorophyll and carotenoids), and stress tolerance [9,10,11,12]. Therefore, the nanoparticle–plant interface forms a dynamic physiological system in which nanomaterials can act as elicitors, growth stimulators, or stressors, depending on their physicochemical properties and the plant’s adaptive capacity.

Metal oxide NPs play an important role in fertilization, gene delivery, and pest control within plant biotechnology [2,13]. Among these, copper oxide (CuO) nanoparticles are commonly used as fertilizers [14], pesticides [15], and elicitors [16]. Copper (Cu) is an essential micronutrient for plants, with different species having varying requirements. Both deficiency and excess of Cu can cause issues such as chlorosis, necrosis, and leaf scorching. Cu plays vital roles in photosynthesis, respiration, enzyme activity, and the biosynthesis of antioxidant enzymes and carotenoids [17,18]. CuO NPs have advantages over bulk Cu as a plant nutrient due to their increased surface area, improved bioavailability, and ability to promote growth at low concentrations, while also serving as a mild stressor that can stimulate secondary metabolism. Supplementing with CuO NPs has been shown to eliminate endophytic bacterial contamination [19], improve callus induction [20], encourage callus growth [21], facilitate somatic embryogenesis [22], enhance shoot regeneration [23], and boost secondary metabolite production [20,21,24,25].

*Ajuga multiflora* Bunge, known as bugleweed, belongs to the Lamiaceae family and serves as an attractive ornamental ground cover and medicinal herb [26]. It is widely distributed in East Asia [27]. *A. multiflora* exhibits various biological activities, including muscle protection, cytotoxic effects against murine leukemia tumors, antibacterial properties, and pesticidal capabilities [28,29,30,31]. It contains several bioactive compounds, for example, 20-hydroxyecdysone, 29-hydroxyprecyasterone, cyasterone, makisterone A, 8-O-acetylharpagide, harpagide, apigenin, apigenin 7-glucuronide, δ-, γ-, and α-tocopherols, (all-*E*)-β-carotene, (all-*E*)-β-cryptoxanthin, (all-*E*)-lutein, 9-*Z*-neoxanthin, (all-*E*)-violaxanthin, (all-*E*)-zeaxanthin, and fatty acids [26,27,29,30,32,33], which may contribute to its therapeutic effects. Due to limited propagation through conventional methods [34,35], in vitro propagation offers an effective pathway for large-scale cultivation [26,32,36]. In vitro cell, organ, and somatic embryo cultures enable metabolite production [26,27,32,37,38]. Several abiotic elicitors, such as coronatine, melatonin, methyl jasmonate, phenyl acetic acid, salicylic acid, and sodium nitroprusside, as well as biotic elicitors like chitosan, pectin, and yeast extract, along with precursors like mevalonate acid, L-phenylalanine, and terpineol, have been used to enhance metabolite production from in vitro cultures of *Ajuga* species, including *A. multiflora* [30,38,39,40,41,42].

Although NPs have been shown to increase secondary metabolite production in several members of the Lamiaceae family [43,44,45,46,47], no studies have explored their effects on secondary metabolites in in vitro cultures of *Ajuga* species. Additionally, micropropagation systems are ideal platforms for studying interactions between nanoparticles and plants, providing controlled environments that support morphogenesis and metabolite biosynthesis [48,49]. Many studies have indicated that incorporating NPs during in vitro culture can influence germination, organogenesis, and rooting in various plants, but research on *Ajuga* species remains limited. However, most plant tissue culture research focuses primarily on short-term NP exposure. Therefore, further investigation is needed into the effects of continuous NP exposure on later stages of in vitro propagation. This study examined the effects of CuO NPs on the in vitro propagation of *A. multiflora*, with a specific focus on adventitious shoot (AS) regeneration, axillary shoot multiplication, rooting, and the synthesis of lipophilic metabolites in regenerated microshoots and plants.

## 2. Results

### 2.1. Effect of CuO NPs on In Vitro Propagation

#### 2.1.1. AS Regeneration from Leaf Explants

**Figure 1:**
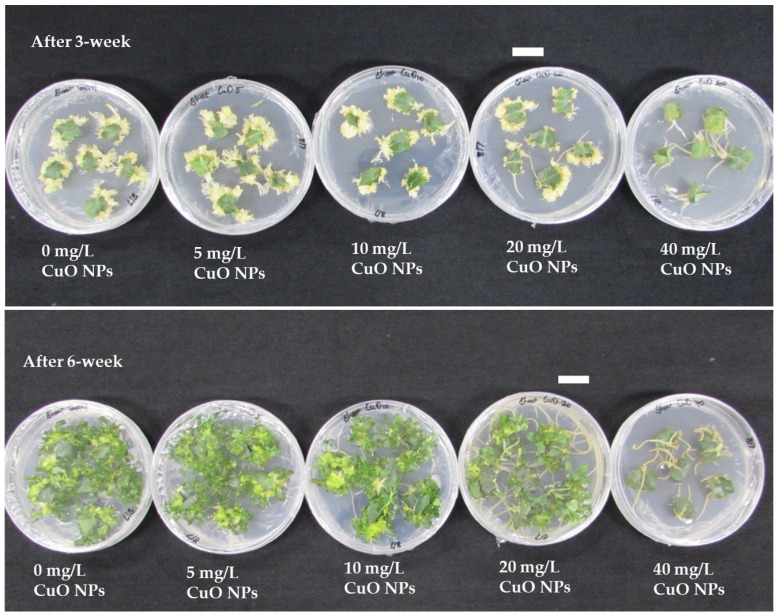
Effect of different concentrations of CuO NPs on direct adventitious shoot regeneration from leaf explants of *A. multiflora*. Bar: 1.0 cm.

Leaf explants of *Ajuga multiflora* readily formed direct AS buds, adventitious roots, or both (Figure 1), depending on CuO NPs levels. The ASI frequency remained unaffected (100%) at concentrations of 0–20 mg/L CuO NPs but sharply declined to 8.3% at 40 mg/L CuO NPs (Table 1). The number of ASs ranging from 1.1 to 29.4 per explant varied significantly among treatments (*p* < 0.0001), with the highest number of ASs (29.4) at 5 mg/L CuO NPs, representing a 24% increase over the control (23.7). Shoot production decreased progressively at higher CuO NPs concentrations, reaching only 1.1 at 40 mg/L CuO NPs (Table 1 and Figure 1). Shoot FW was also significantly influenced by CuO NPs. While 0–10 mg/L showed no significant difference from the control, FW increased markedly at 20 mg/L (0.151 g FW), 44% higher than the control (0.105 g FW). However, 40 mg/L suppressed shoot biomass to just 0.013 g. In addition, explants cultured at 20 and 40 mg/L CuO NPs displayed root emergence, while at 40 mg/L, rhizogenesis dominated with complete suppression of AS induction (Figure 1).

#### 2.1.2. ASM from Shoot Tips

**Figure 2:**
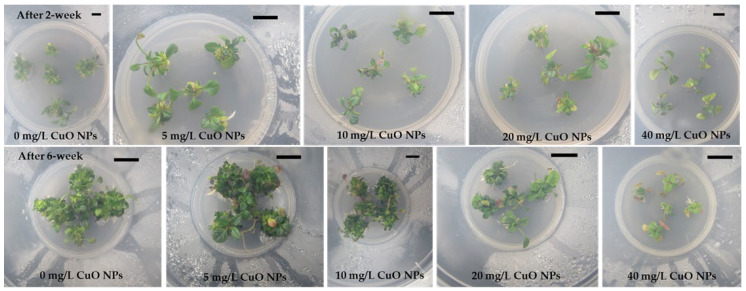
Effect of different concentrations of CuO NPs on axillary shoot multiplication from shoot tip explants of *A. multiflora*.

Shoot tips excised from regenerated ASs were further cultured to evaluate the effect of CuO NPs levels on ASM (Table 2). After 2 weeks of incubation, shoot tip explants produced multiple shoots; however, the explants failed to develop multiple shoots on MS medium containing 40 mg/L CuO NPs (Figure 2). The shoot induction frequency remained at 100% up to 20 mg/L CuO NPs but decreased to 71.1% at 40 mg/L (Table 2 and Figure 2). The highest ASM was achieved at 5 mg/L (22.4 shoots/explant), which is 29% higher than the control (17.3). Conversely, the number of shoots declined at 10 mg/L (15.9) and 20 mg/L (9.6) and was nearly eliminated at 40 mg/L (1.0). Shoot FW reached its peak at 10 mg/L (0.269 g FW), significantly exceeding the control (0.211 g FW). Moderate FW values persisted at 5 and 20 mg/L, but 40 mg/L markedly reduced biomass (0.106 g FW).

#### 2.1.3. Rooting and Plantlet Development

**Figure 3:**
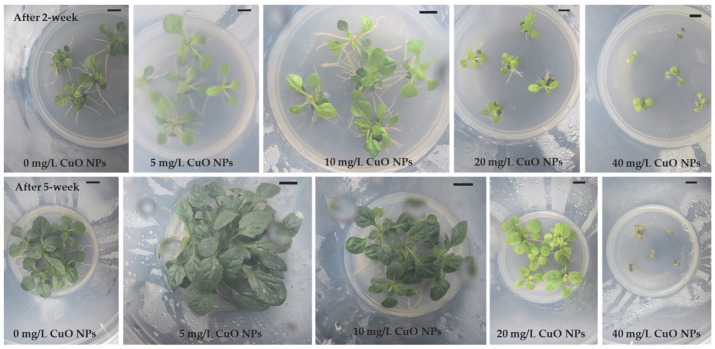
Effect of different concentrations of CuO NPs on rooting of *A. multiflora*.

CuO NPs also significantly influenced rhizogenesis and plantlet growth (Table 3). After two weeks of incubation, the explants developed adventitious roots except for those on RI medium with 40 mg/L CuO NPs (Figure 3). The continuous exposure to higher concentrations resulted in severe growth inhibition and, ultimately, death of cultured shoots, indicating a toxic effect of CuO NPs on rooting. Morphological alterations, including reduced shoot elongation, leaf yellowing, and suppressed root formation, were evident at elevated nanoparticle levels (Figure 3). The RI frequency remained at 100% in the 0–10 mg/L CuO NPs treatments but declined to 80.6% at 20 mg/L CuO NPs and was completely inhibited at 40 mg/L CuO NPs. The maximum average root number was observed at 10 mg/L CuO NPs, with 11.1 roots per shoot, and at 5 mg/L, with 10.2 roots per shoot. Both values were significantly higher than the control group, which had an average of 5.7 roots. However, the average root number decreased to 4.8 at 20 mg/L, and rooting was absent at 40 mg/L. Plantlet FW exhibited the highest value at 5 mg/L (2.26 g FW), representing a 32% increase compared to the control (1.71 g FW). However, biomass declined significantly at 10 mg/L (1.35 g FW) and sharply decreased at 20 mg/L (0.52 g FW), with complete inhibition at 40 mg/L.

### 2.2. Effect of CuO NPs on Lipophilic Metabolites

#### 2.2.1. Impact of CuO NPs Levels on α-Tocopherol and Carotenoid Contents in Microshoot and Leaf Tissues of *A. multiflora*

**Figure 4:**
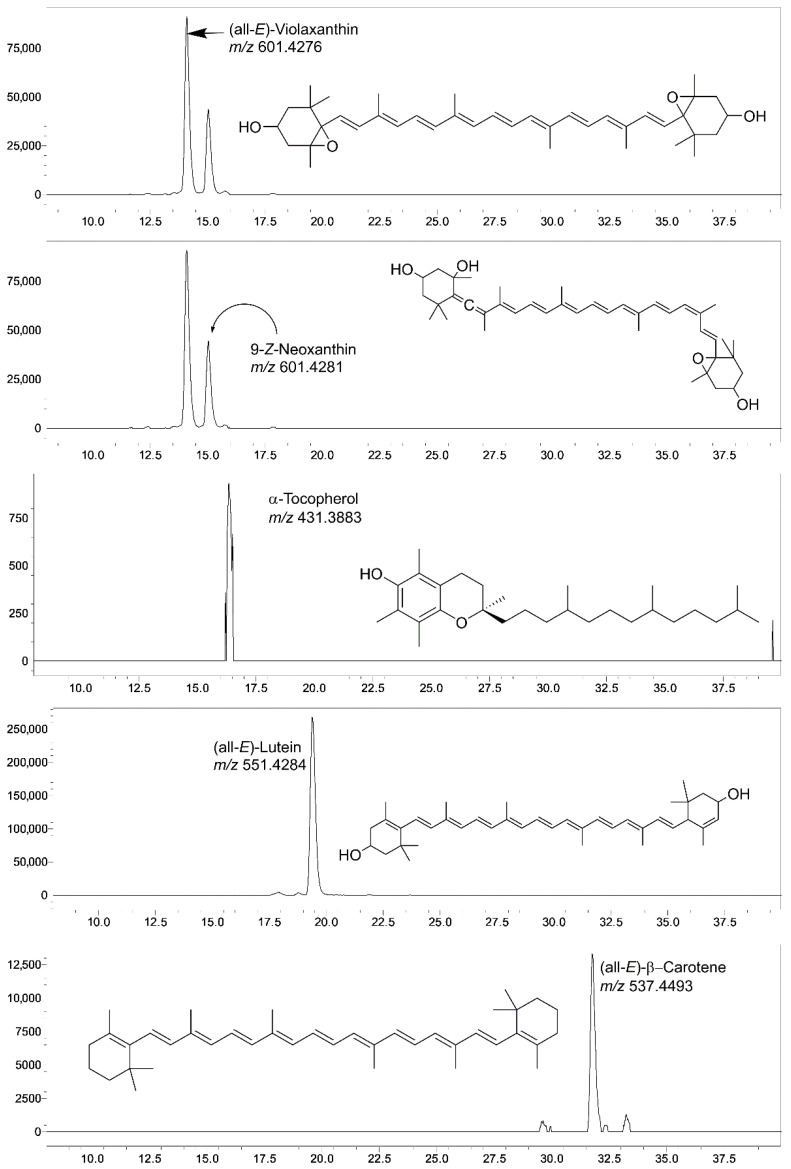
Liquid chromatography (LC)-single ion monitoring (SIM)–mass spectrometry (MS) chromatograms of (all-*E*)-violaxanthin, 9-*Z*-neoxanthin, α-tocopherol, (all-*E*)-lutein, and (all-*E*)-β-carotene identified in *A. multiflora*.

The LC-SIM-based MS method employed for the identification and quantification of carotenoids and tocols in microshoots and leaf tissues of *A. multiflora* revealed the presence of significant amounts of (all-*E*)-violaxanthin, 9-*Z*-neoxanthin, α-tocopherol, (all-*E*)-lutein, and (all-*E*)-β-carotene (Figure 4).

**Figure 5:**
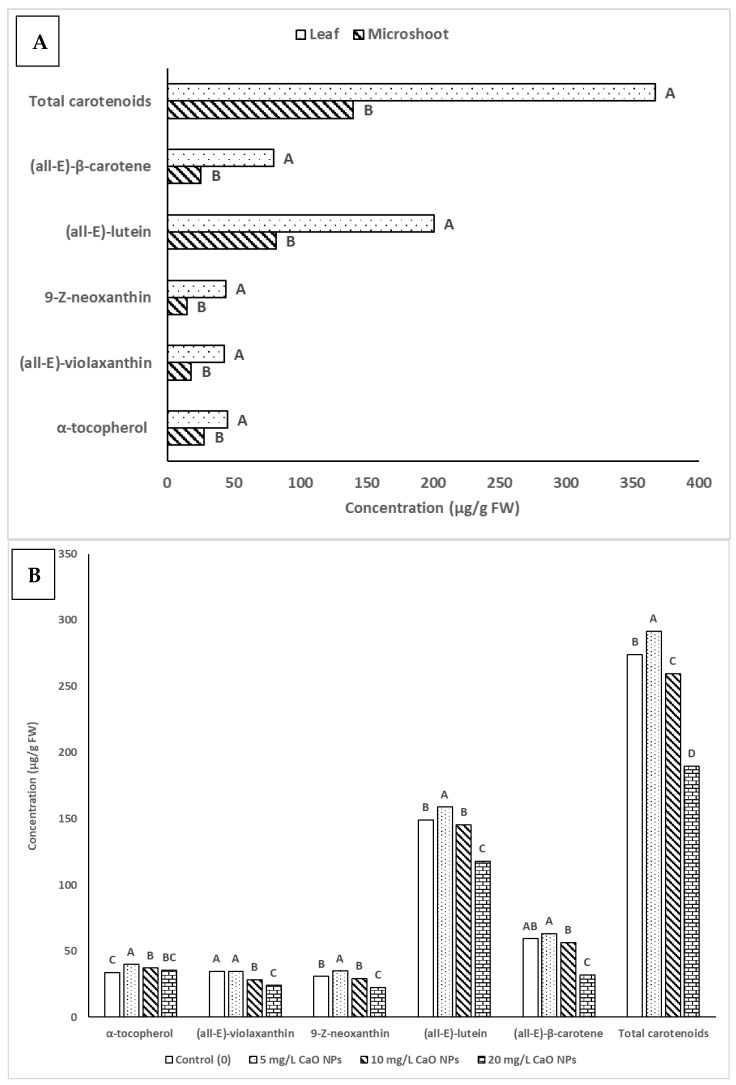
Impact of organ type (**A**) and CuO NPs concentration (**B**) on the contents of α-tocopherol and carotenoids. Different uppercase letters (A–D) in the bar were significantly different at *p* < 0.05 according to DMRT.

The levels of α-tocopherol and individual carotenoids were significantly affected by CuO NPs concentrations, organ type (microshoots and leaves), and their interactions (Org*Con) (Table 4). In microshoots, α-tocopherol was 26.5 µg/g FW in control, slightly rising at 5 and 10 mg/L CuO NPs, but remaining statistically similar; at 20 mg/L CuO NPs, it was 44.6 µg/g FW. Carotenoids like (all-*E*)-violaxanthin and 9-*Z*-neoxanthin increased at 20 mg/L CuO NPs. Total carotenoids peaked at 5 mg/L (51.6 µg/g FW), the lowest in control. In leaves, α-tocopherol levels increased from 41.2 to 50.3 µg/g FW at 5 mg/L CuO NPs, then slightly decreased. Carotenoids like (all-*E*)- (all-*E*)-violaxanthin and (all-*E*)-lutein also peaked at 5 mg/L CuO NPs. Total carotenoids were highest at 5 mg/L CuO NPs (447.1 µg/g FW) (Table 4). Leaves accumulated significantly higher levels of lipophilic metabolites than microshoots (Figure 5A). α-tocopherol content in leaves was 45.4 µg/g FW, compared to 27.8 µg/g FW in microshoots. Similarly, (all-*E*)-lutein level was higher in leaves (200.6 µg/g FW) than in microshoots (81.9 µg/g FW). The concentration of CuO NPs affected metabolite accumulation (Figure 5B): at 5 mg/L, α-tocopherol (39.8 µg/g FW) and total carotenoids (291.4 µg/g FW) increased relative to the control. However, at 10 mg/L and 20 mg/L CuO NPs, α-tocopherol decreased to 37.1 µg/g FW and 35.6 µg/g FW, respectively. (all-*E*)-violaxanthin content was highest in the control (34.6 µg/g FW) but declined with increasing CuO NPs concentrations, while 9-*Z*-neoxanthin peaked at 5 mg/L (34.8 µg/g FW) before decreasing. (all-*E*)-lutein content reached its peak at 5 mg/L CuO NPs, measuring 158.8 µg/g FW, while the control group had a slightly lower level of 149.1 µg/g FW. It decreased at 10 mg/L CuO NPs (145.4 µg/g FW) and dropped significantly at 20 mg/L CuO NPs (117.7 µg/g FW). (all-*E*)-β-carotene was highest at 5 mg/L CuO NPs (63.1 µg/g FW), with the control close behind (59.2 µg/g FW), then decreased at 10 mg/L (56.3 µg/g FW) and sharply at 20 mg/L CuO NPs (31.7 µg/g FW) (Table 4).

#### 2.2.2. Impact of CuO NPs Levels on Phytosterols Content in Microshoot and Leaf Tissues of *A. multiflora*

**Figure 6:**
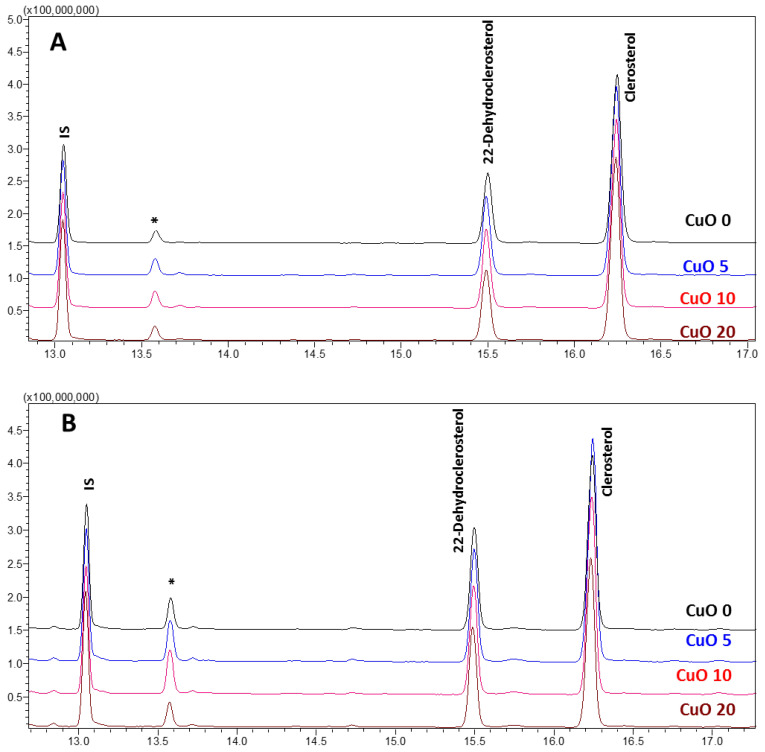
GC-MS total ion chromatograms (TICs) of *A. multiflora*. (**A**) Microshoots, (**B**) leaves; IS: internal standard (5β-Cholestan-3α-ol). * α-tocopherol. CuO 0, 5, 10, and 20 represent application of CuO nanoparticles (NPs) at the concentrations of 0, 5, 10, and 20 mg/L, respectively.

**Figure 7:**
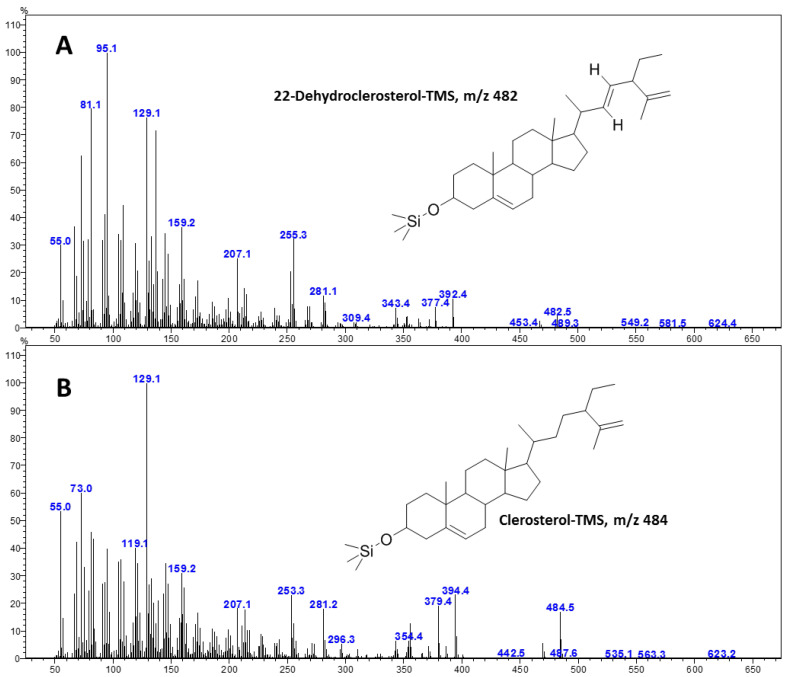
Mass spectrum of 22-Dehydroclerosterol-trimethylsilyl (TMS) derivative (**A**) and clerosterol-trimethylsilyl (TMS) derivative (**B**) identified in *A. multiflora*.

**Figure 8:**
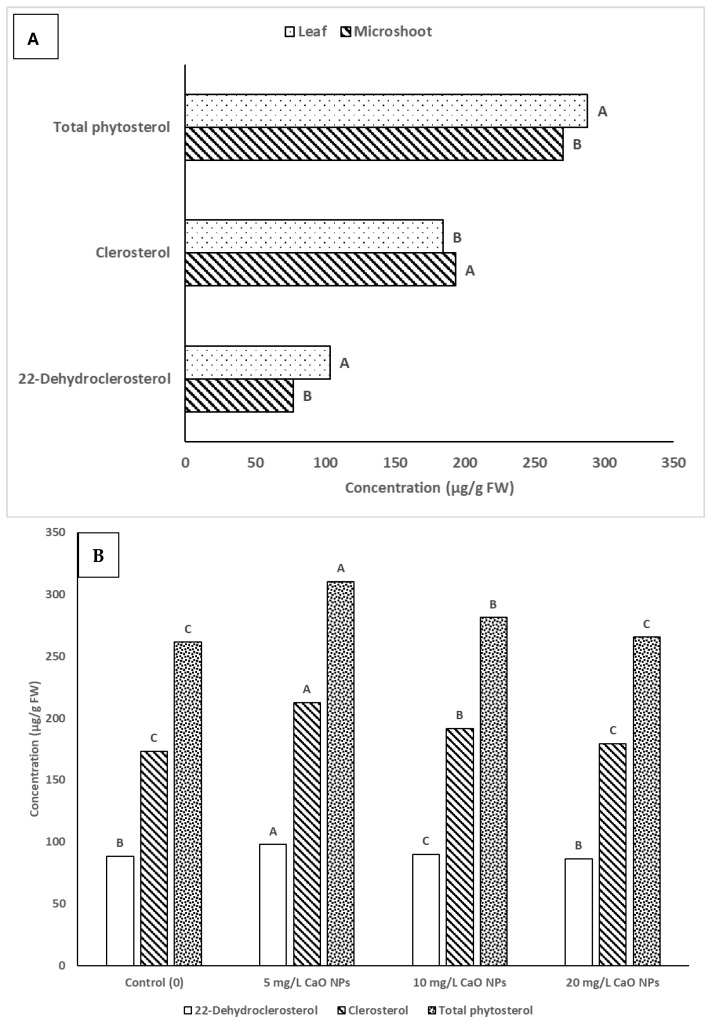
Impact of organ type (**A**) and CuO NPs concentration (**B**) on the content of sterols. Different uppercase letters (A–C) in the bar were significantly different at *p* < 0.05 according to DMRT.

The GC-MS method used to identify and quantify phytosterols in *A. multiflora* microshoot and leaf tissues indicated high levels of 22-dehydroclerosterol and clerosterol (Figure 6). The presence of these compounds in *A. multiflora* microshoots and leaf tissues was verified by their MS fragmentation pattern (Figure 7). The levels of these phytosterols were significantly affected by CuO NPs concentrations, organ type (microshoots and leaves), and their interactions (Org*Con). Among the treatments, leaves exhibited higher levels of 22-dehydroclerosterol, ranging from 99.4 to 108.5 µg/g FW, compared to microshoots, which contained 71.2 to 86.8 µg/g FW. Conversely, the content of clerosterol was marginally greater in microshoots (168.3 to 214.4 µg/g FW) than in leaves (168.6 to 210.5 µg/g FW) (Table 5). Consequently, the total phytosterol concentration was moderately higher in leaves (288.2 µg/g FW) than in microshoots (270.8 µg/g FW) (Figure 8A). The supplementation of CuO NPs demonstrated a concentration-dependent effect on sterol accumulation (Figure 8B). At 5 CuO NPs mg/L, the levels of 22-dehydroclerosterol (97.6 µg/g FW), clerosterol (212.4 µg/g FW), and total phytosterols (310.1 µg/g FW) significantly increased compared to control values (88.3, 172.9, and 261.3 µg/g FW, respectively). This increase represents the maximum accumulation recorded in the present study. However, higher concentrations (10–20 mg/L CuO NPs) led to a gradual decline in sterol levels. At 20 mg/L CuO NPs, the total phytosterols decreased to 265.4 µg/g FW, returning to levels comparable to those of the control (Figure 8B).

#### 2.2.3. Impact of CuO NPs Levels on Fatty Acids Content in Microshoot and Leaf Tissues of *A. multiflora*

The content of fatty acids was significantly influenced by CuO NPs concentrations, organ type (microshoots and leaves), and their interactions (Org*Con) (Table 6). In microshoots, palmitic acid content was highest at 0 mg/L CuO NPs (21.7%) but decreased at higher concentrations, reaching 19.0% at 20 mg/L. Stearic acid remained stable (3.7–3.8%), while oleic acid peaked at 10.7% at 5 mg/L CuO NPs and then dropped to 7.1% at 20 mg/L. Linoleic acid increased from 25.7% to 28.3% at 20 mg/L CuO NPs, and α-linolenic acid peaked at 40.6% at that concentration. Total saturated fatty acids (SFAs) decreased from 26.6% to 23.9%, while total polyunsaturated fatty acids (PUFAs) rose from 65.2% to 69.0%. The (omega-6) n-6/n-3 PUFAs ratio remained stable. For leaves, palmitic acid started lower at 11.2% but increased to 18.8% at 20 mg/L CuO NPs. Stearic acid increased from 1.8% to 3.9%. Oleic acid reached 13.6% at the highest concentration. Linoleic acid fluctuated, decreasing to 17.0% at 10 mg/L CuO NPs but increasing again to 21.7% at 20 mg/L. α-Linolenic acid dropped from 61.6% to 41.7%. Total SFAs increased from 13.2% to 23.3%, while total PUFAs decreased from 80.3% to 63.4%. The n-6/n-3 ratio increased from 0.30 to 0.52 (Table 6).

**Figure 9:**
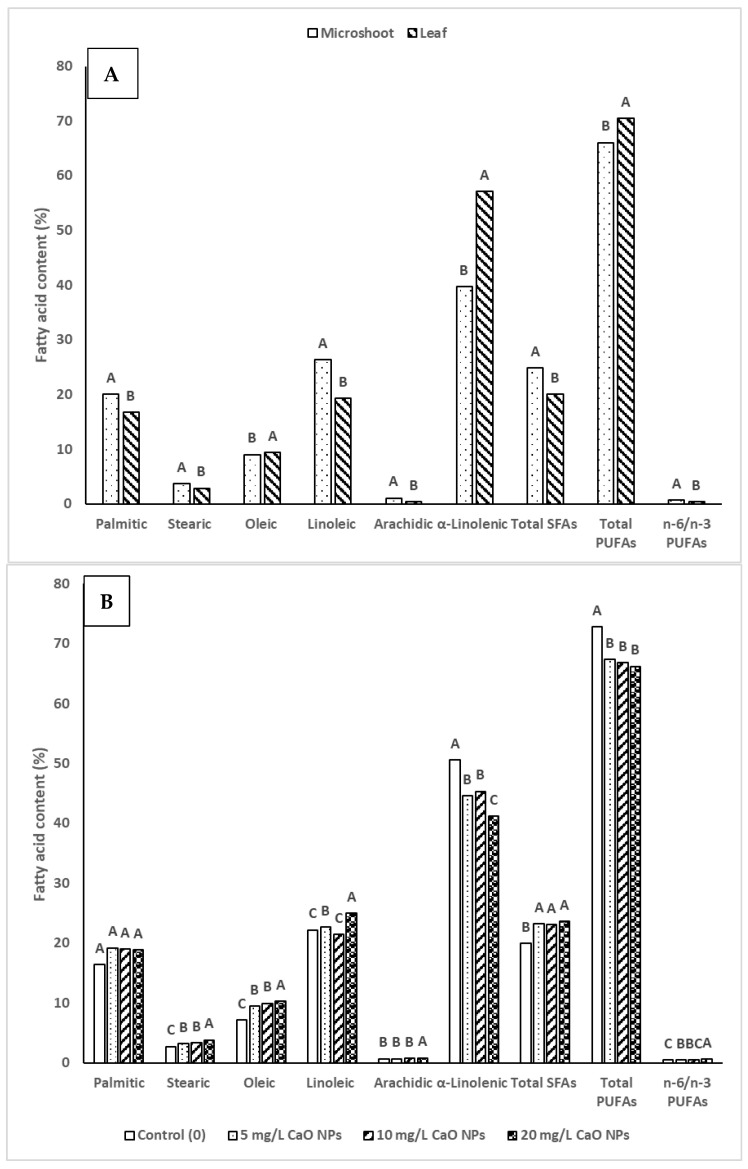
Impact of organ type (**A**) and CuO NPs concentration (**B**) on the content of fatty acids. Different uppercase letters (A–C) in the bar were significantly different at *p* < 0.05 according to DMRT.

Microshoots contained higher levels of SFAs, while leaves accumulated more PUFAs (Figure 9A). For example, palmitic acid was notably higher in microshoots (20.0 µg/g FW) compared to leaves (16.7 µg/g FW), whereas α-linolenic acid was nearly 1.5 times greater in leaves (57.1 µg/g FW) than in microshoots (39.6 µg/g FW). Consequently, the n-6/n-3 PUFAs ratio was significantly higher in microshoots (0.67) than in leaves (0.39), indicating a more balanced fatty acid profile in leaf tissues (Figure 9A). CuO NPs supplementation showed a concentration-dependent effect on fatty acid metabolism (Figure 9B). At 5–10 mg/L CuO NPs, oleic acid (9.49–9.98 µg/g FW) and total SFAs (23.1 µg/g FW) increased significantly compared with the control (7.21 µg/g FW and 19.93 µg/g FW, respectively). However, α-linolenic acid decreased at these concentrations (44.58–45.36 µg/g FW) compared to control levels (50.5 µg/g FW). At 20 mg/L CuO NPs, linoleic acid reached the maximum (24.9 µg/g FW), but α-linolenic acid fell to the lowest level (41.1 µg/g FW), resulting in the highest n-6/n-3 PUFAs ratio (0.61).

## 3. Discussion

Although the beneficial effects of various NPs on plant cells, tissues, and organs in vitro cultures have been documented in different plant species, their effects on *Ajuga* species have not been disclosed. Among the various NPs, supplementing metal oxide NPs to plant tissue culture media has been found to eliminate microbial contamination, enhance the morphogenic potential of explants, and boost bioactive compound production through cell and organ cultures [49]. CuO NPs are one of the most economically feasible nanomaterials when compared with other metal oxide nanoparticles, and they have been reported to enhance callus induction, organogenesis, and somatic embryogenesis [50]. However, the impact of CuO NPs on direct adventitious shoot regeneration has not been documented. The present study evaluated the effect of CuO NPs on ASI from leaf explants of *A. multiflora*. The results showed that CuO NPs influenced the direct ASI of *A. multiflora* leaf explants in a concentration-dependent manner. The highest number of AS was obtained when the MS medium was supplemented with 5 mg/L CuO NPs. However, higher levels of CuO NPs (20 or 40 mg/L) decreased AS production and promoted root induction (Table 1, Figure 1). These results highlight a biphasic effect of CuO NPs on adventitious organogenesis: low concentrations promoted ASI, while high concentrations, particularly at 40 mg/L CuO NPs, shifted development toward adventitious root induction. Adventitious shoots or roots form from the cut ends of the *A. multiflora* explants (Figure 1). Thus, the cells at the cut end that come into contact with CuO NPs may be exposed to different levels of Cu. This variable exposure can cause different stress responses within the cells, which may significantly affect the induction of shoots or roots. In response to such stress, plant cells often activate specific pathways that enable adaptive growth responses. Therefore, it is important to consider how interaction with CuO NPs can influence cellular behavior and subsequent plant development. Understanding these mechanisms is vital for harnessing the potential of CuO NPs in plant tissue culture. Zafar et al. [51] reported that leaf and stem explants of *Brassica nigra* cultured on CuO NPs (1–20 mg/L) containing medium showed different responses: lower concentrations of CuO NPs stimulated root formation from callus, while higher concentrations, 10 and 20 mg/L, caused roots to emerge directly from the explants. CuO NPs may produce reactive oxygen species and disrupt phytohormonal balance, leading to biphasic effects [21,52]. At lower levels of CuO NPs, we can speculate that moderate production of reactive oxygen species may serve as a signaling mechanism. This could activate antioxidant enzymes like catalase, peroxidases, and superoxide dismutase, which might help maintain ion balance and influence phytohormone levels such as auxin and cytokinin, possibly enhancing morphogenic ability. However, it is worth considering that if CuO NPs levels exceed a plant’s buffering capacity, reactive oxygen species could shift from a regulatory role to a harmful one. This shift might lead to membrane damage and oxidative stress, affecting proteins, lipids, and nucleic acids, and potentially causing hormonal imbalances. Such changes could decrease biomass and increase phytotoxicity [8,9,10,11]. This proposed hormesis-based biphasic response suggests that the impact of CuO NPs is not strictly toxic but depends on dosage, transitioning from adaptive stress responses at lower levels to detrimental oxidative and hormonal effects at higher concentrations. Further investigation would be needed to gather data to support these ideas.

The ability of shoot tips isolated from ASs developed on CuO NPs medium was evaluated for ASM. Results showed that the addition of low CuO NP concentrations (5–10 mg/L) supports ASM, either by increasing shoot number or shoot FW, while higher concentrations decrease both parameters. The findings are consistent with the ASI stage, confirming that CuO NPs affect morphogenesis in a dose-dependent manner. The rate of shoot induction also significantly decreased when *A. multiflora* shoot tips were cultured on 40 mg/L CuO NPs. The findings are consistent with those of Javed et al. [25], which indicated that the maximum shoot length and FW of *Stevia rebaudiana* were observed on a medium containing 10 mg/L CuO NPs. Conversely, the percentage of shoot induction diminished when nodal explants of *S. rebaudiana* were cultured on media with elevated concentrations of CuO NPs ranging from 100 to 1000 mg/L. According to Balamurugan et al. [53], there are two primary ways in which CuO NPs contribute to enhanced regeneration in plants. Firstly, Cu is a vital nutrient that supports numerous physiological functions. It triggers the activity of enzymes that are important for lignin production and are essential for various enzyme systems. Secondly, Cu is crucial for fundamental plant processes, including photosynthesis, respiration, and the metabolic pathways of carbohydrates and proteins.

The ability of shoot tips isolated from axillary shoots grown on CuO NPs medium was evaluated for RI. Lower concentrations (5 and 10 mg/L) increased root number and plantlet FW, while higher concentrations (20 and 40 mg/L) significantly inhibited root induction, root number, and plantlet FW, with complete inhibition at 40 mg/L. Leaf chlorosis was also observed in the 20 mg/L CuO NPs treatment. These findings suggest that, although CuO NPs may promote rooting at lower doses, their accumulation causes phytotoxic effects, damaging both shoot survival and RI. Fedorova et al. [54] reported that shoots of poplar and downy birch produced more roots when the medium was supplemented with 5 mg/L CuO NPs. However, the root induction percentage decreased in both species. In contrast, CuO NPs treatment did not improve in vitro rooting of red oak [55]. On the other hand, seedlings of *Brassica juncea* cultured on the MS medium with 20–500 mg/L CuO NPs showed reductions in shoot length, total chlorophyll, and carotenoids [56]. In maize, seedlings exposed to 10–100 mg/L CuO NPs exhibited chlorotic symptoms in their third leaves and had reduced biomass [57]. Elevated Cu levels can harm cell walls, membranes, mitochondria, chloroplasts, and lysosomes and can increase reactive oxygen species levels, leading to cell death [58,59,60,61].

Nano-elicitation is one of the top strategies worldwide for achieving sustainable and consistent production of bioactive compounds [21]. Several studies have shown that supplementing plant cell and organ cultures with NPs often boosts secondary metabolite production. Among the NPs, metal oxide NPs play dual roles in enhancing the production of industrially important metabolites through plant cells or organ cultures by serving not only as elicitors but also as nutrients [2,48]. The inclusion of CuO NPs in in vitro culture media increased the production of total phenolic content in the callus of *Stevia rebaudiana* [25], *Solanum nigrum* [24], *Vigna radiata* [62], as well as chicoric acid, eugenol, rosmarinic acid, total phenolics, and flavonoids in the callus of *Ocimum basilicum* [21]. Similarly, the addition of CuO NPs enhanced the production of rebaudioside A, stevioside, total phenolics, and flavonoids in shoot cultures of *Stevia rebaudiana* [25]. Recently, Bamal et al. [63] reported the effect of CuO NPs on the production of both primary (amino acids, starch, sugar, and protein) and secondary (total phenolics, flavonoids, and lupeol) metabolites in in vitro cultures of *Alhagi maurorum*. The impact of copper oxide nanoparticles (CuO NPs) on the production of lipophilic compounds, such as carotenoids, fatty acids, phytosterols, and tocopherols in plant cell or organ cultures, remains inadequately explored. In this study, we investigate the effects of CuO NPs on the biosynthesis of α-tocopherol, carotenoids, sterols, and fatty acids in *A. multiflora*. The results indicate that the modulation of these compounds occurs in a concentration- and organ-dependent manner.

The stimulatory effect of CuO NPs on carotenoid synthesis in leaves was especially noticeable, with (all-*E*)-violaxanthin, 9-*Z*-neoxanthin, and (all-*E*)-lutein reaching 51.6, 54.2, and 238.7 µg/g FW, respectively, at 5 mg/L, the highest values recorded in this study (Table 4). However, at 20 mg/L, the total carotenoid content in leaves dropped to 226.5 µg/g FW, nearly half of the maximum observed at 5 mg/L. In contrast, microshoots showed a moderate increase in (all-*E*)-violaxanthin (21.1 µg/g FW) and (all-*E*)-lutein (90.6 µg/g FW) at 20 mg/L. Conversely, the same concentration caused sharp reductions in 9-*Z*-neoxanthin (27.3 µg/g FW) and (all-*E*)-β-carotene (39.4 µg/g FW) in leaves. This difference suggests that metabolic regulation under nanoparticle stress varies by organ, with leaves being more sensitive to high CuO NP exposure. The increase in α-tocopherol content in both organs at 5 mg/L CuO NPs observed here may indicate activation of protective mechanisms against mild oxidative stress, while the decrease at 20 mg/L suggests that toxicity surpasses the plant’s protective threshold (Table 4). The stimulatory effect at 5 mg/L CuO NPs suggests potential for controlled use to enhance antioxidant metabolite production, while the inhibitory effects at higher concentrations emphasize the need for dosage optimization. Likewise, the carotenoid content in *Brassica juncea* seedlings decreased when treated with 20–500 mg/L CuO NPs [56]. Previous studies have also reported that nanoparticles at low doses can stimulate antioxidant defenses, while excessive exposure disrupts chloroplast integrity and pigment biosynthesis.

In the context of phytosterols, clerosterol reached 214.4 µg/g FW in microshoots at 5 mg/L, the highest among all treatments, while in leaves, the same concentration increased both 22-dehydroclerosterol (108.5 µg/g FW) and total phytosterols (319.1 µg/g FW). However, at 20 mg/L, reductions were more significant in leaves (total sterols: 268.0 µg/g FW) than in microshoots (262.9 µg/g FW). These results also suggest that leaf tissues are more sensitive to CuO NP toxicity. Low levels of CuO NPs appear to act as elicitors, stimulating sterol biosynthesis, while higher concentrations may impair isoprenoid metabolism due to oxidative stress or nanoparticle-induced membrane disruption.

For fatty acids, linoleic acid peaked at 28.3 µg/g FW in microshoots at a concentration of 20 mg/L CuO NPs. In contrast, leaves showed the highest α-linolenic acid content (61.6 µg/g FW) in the control group, but this sharply declined with increasing CuO NP concentration, reaching only 41.7 µg/g FW at 20 mg/L. This suggests that leaves are more sensitive to CuO NP-induced changes in unsaturated fatty acid metabolism compared to microshoots. While leaves tend to accumulate PUFAs, they are also more vulnerable to CuO NP toxicity. On the other hand, microshoots maintain relatively stable fatty acid profiles. The positive effects observed at concentrations of 5–10 mg/L indicate potential for eliciting certain fatty acids, but the detrimental impact seen at 20 mg/L highlights the need for careful dosage optimization.

## 4. Materials and Methods

### 4.1. Impact of CuO NPs on Micropropagation

#### 4.1.1. Plant Materials

Healthy young leaves were harvested from actively growing *A. multiflora* plants cultivated in a greenhouse. The leaves were surface disinfected with ethanol (70%, for 90 s), sodium hypochlorite (1%, for 10 min), and mercuric chloride (0.1%, for 6 min). Each treatment was followed by 3–4 rinses (90–100 s per rinse) with sterile deionized water. The explants (0.5–0.7 cm) were prepared from disinfected leaves and placed on a Petri dish containing basal medium (BM) with 6-benzyladenine (BA, 2.0 mg/L) and α-naphthaleneacetic acid (NAA, 0.5 mg/L). The BM consists of Murashige and Skoog [64] nutrients and vitamins, sucrose (3%), and plant agar (0.8%). The pH of the medium (5.60 ± 0.05) was adjusted with potassium hydroxide (0.1 M) and autoclaved (121 °C for 15 min). Petri dishes were maintained at 25 ± 1 °C in darkness for 21 days, then transferred to a 16 h photoperiod provided by a cool white fluorescent tube light (CWFTL) with a photosynthetic photon flux density (PPFD) of 40 µmol s^−1^ m^−2^ for another 21 days. The regenerated shoots were transferred to BM and exposed to a 16 h photoperiod (PPFD of 60–40 µmol s^−1^ m^−2^, CWFTL). The shoots were subcultured onto BM each month. Three-month-old shoots served as the explant source.

#### 4.1.2. Adventitious Shoot Induction (ASI)

Leaf segments (0.5–0.7 cm) were placed on an ASI medium (BM + 2.0 mg/L BA + 0.5 mg/L NAA) supplemented with 0, 5, 10, 20, or 40 mg/L CuO NPs (Copper(II) oxide, <50 nm particle size, Sigma-Aldrich, Saint Louis, MO, USA). The cultures were initially kept in darkness for three weeks, followed by another three weeks under a 16 h photoperiod (40 µmol s^−1^ m^−2^ PPFD using CWFTL) at 25 ± 1 °C. Each treatment consisted of six explants with ten replicates. The percentage of ASI, number of shoots, and shoot fresh weight (FW, mg) were recorded after six weeks of culture.

#### 4.1.3. Axillary Shoot Multiplication (ASM)

Shoot tips separated from shoots induced by 0–40 mg/L CuO NPs treatments were cultured on the same fresh medium for ASM. For the 40 mg/L CuO NPs treatment, the shoots regenerated from an ASI medium containing 20 mg/L CuO NPs served as the source of explants. Each treatment consisted of five explants with ten replicates. The percentage of SI, number of shoots, and shoot FW (mg) were recorded after six weeks of culture.

#### 4.1.4. Root Induction (RI)

Shoot tips separated from the axillary shoot cluster induced by 0–40 mg/L CuO NPs treatments were cultured on the same fresh medium for RI. For the 40 mg/L CuO NPs treatment, the axillary shoots induced from an ASM medium containing 20 mg/L CuO NPs served as the source of explants. Each treatment consisted of five explants with ten replicates. The percentage of RI, number of roots, and plantlet FW (mg) were recorded after five weeks of culture.

### 4.2. Impact of CuO NPs on the Content of Lipophilic Compounds

#### 4.2.1. Reagents and Standards

Authentic standards of Fatty Acid Methyl Esters (FAME) mix (CRM47885), β-sitosterol (24α-ethyl cholesterol), 5-β-cholestan-3α-ol (epicoprostanol); internal standard (IS), butylated hydroxytoluene (BHT), (all-*E*)-β-carotene, and N,O-bis(trimethylsilyl)trifluoroacetamide (BSTFA) containing 1% trimethylchlorosilane (TMCS), were obtained from Merck Ltd., Seoul, Republic of Korea. A tocols mixture solution comprising α-, β-, γ-, and δ-tocopherol as well as α-, β-, γ-, and δ-tocotrienol was procured from ChromaDex, Inc. (Irvine, CA, USA). The carotenoids (all-*E*)-lutein, 9-*Z*-neoxanthin, and (all-*E*)-violaxanthin, employed in this study, were isolated from lettuce following a previously established purification protocol [65]. All organic solvents utilized for extraction procedures were of liquid chromatography (LC) grade and were sourced from J.T. Baker^®^ (Avantor Korea, Suwon-Si, Republic of Korea).

#### 4.2.2. Extraction of Lipophilic Compounds (Crude Lipids)

The microshoots and leaves of *A. multiflora* were collected from 0 to 20 mg/L CuO NPs treatment. Due to insufficient sample quantity, the 40 mg/L treatment was not determined, as the explants did not survive under this condition. Lipophilic compounds, including tocols, carotenoids, fatty acids, and sterols (phytosterols), were concurrently extracted from microshoots collected from ASI media and leaves from regenerated plantlets grown in RI media using an optimized protocol previously established by Kim et al. [66], with slight modifications. A comprehensive description of the extraction procedure is provided in Figure S1. To inhibit the oxidative degradation of lipophilic compounds during extraction, the synthetic antioxidant butylated hydroxytoluene (BHT) was incorporated into the extraction solvent at a concentration of 0.1% (*w*/*v*) as suggested by Saini and Keum [67].

Tocols and carotenoids were concurrently analyzed using liquid chromatography (LC) coupled with selected-ion monitoring (SIM)-based mass spectrometry (MS) techniques, omitting hydrolysis to prevent degradation of these lipophilic phytochemicals [68]. A portion of the extracted crude lipids was subjected to hydrolysis and subsequently converted into fatty acid methyl esters (FAMEs) (refer to Figure S2) and trimethylsiloxy [–O-Si(CH_3_)_3_; TMS] derivatives (refer to Figure S3) following the methodology described by [68]. These derivatives were then employed for the analysis of fatty acids and phytosterols using gas chromatography with flame ionization detection (GC-FID) and gas chromatography-mass spectrometry (GC-MS), respectively.

#### 4.2.3. LC-SIM-MS Analysis of Carotenoids and Tocols

The major carotenoids and tocols were quantitatively analyzed utilizing a Liquid Chromatography (LC)–Selected Ion Monitoring (SIM) based Mass Spectrometry (MS) approach, employing the LCMS-9030 quadrupole time-of-flight (Q-TOF) mass spectrometer (Shimadzu, Tokyo, Japan). The LC-MS conditions applied for the analysis of carotenoids and tocols are detailed in Table S1. Additionally, the Selected Ion Monitoring (SIM) transition (*m*/*z*) utilized for the identification and analysis of carotenoids and tocols were as follows: α- tocopherol (*m*/*z* of 431.3883), β- and γ-tocopherol (*m*/*z* of 416.3669), δ-tocopherol (*m*/*z* of 402.3488), α-tocotrienol (*m*/*z* of 425.3423), β- and γ-tocotrienol (*m*/*z* of 411.3268), δ-tocotrienol (*m*/*z* of 397.3113), (all-*E*)-β-carotene (*m*/*z* of 537.4493), (all-*E*)-lutein (*m*/*z* of 551.4284), 9-*Z*-neoxanthin (*m*/*z* of 601.4281), and (all-*E*)-violaxanthin (*m*/*z* of 601.4276). The limits of detection (LOD) and limits of quantitation (LOQ) were established based on a signal-to-noise (S/N) ratio exceeding 3 and 10, respectively, for quantitative analysis employing LC-MS [69]. Every individual sample was analyzed in four replicates.

#### 4.2.4. Qualitative Analysis of FAMEs Utilizing GC-FID and GC-MS

Fatty acid methyl esters (FAMEs) were qualitatively analyzed using an Agilent 7890B gas chromatograph (GC) equipped with a Flame Ionization Detector (FID; Agilent Technologies Canada, Inc., Mississauga, ON, Canada). The specific analytical parameters employed are detailed in Table S2. For precise identification of FAMEs, mass spectra were obtained utilizing a QP2010 SE gas chromatography-mass spectrometry system (GC-MS; Shimadzu, Japan) in accordance with the GC-FID thermal program. The resulting mass fragmentation patterns were compared against authentic standards and reference spectral libraries, including NIST08S, NIST08, and Wiley9, to verify the identities of the FAME compounds. Every individual sample was analyzed in four replicates.

#### 4.2.5. Qualitative Analysis of Sterols Utilizing GC-MS

Sterols (phytosterols) were analyzed following the silylation of extracted crude lipids using a QP2010 SE GC-MS system (Shimadzu, Tokyo, Japan). The analytical parameters employed are detailed in Table S3. Sterol identification was validated by comparing their mass fragmentation patterns with those of authentic standards and reference databases, including Wiley9, NIST08, and NIST08S. The recoveries of phytosterols were precisely monitored and normalized utilizing 5β-cholestan-3α-ol as the internal standard (IS). Clerosterol and 22-Dehydroclerosterol were quantified using the external calibration curve of β-Sitosterol. Every individual sample was analyzed in four replicates.

### 4.3. Statistical Analysis

All experiments were conducted using a completely randomized design. The data were represented as mean ± standard deviation (S.D.). A one-way analysis of variance (ANOVA) was conducted utilizing IBM SPSS Statistics (version 26), with a significance threshold (*p*) set at 0.05 (Tukey Honestly Significant Difference (HSD)).

## 5. Conclusions

In summary, CuO NPs have significant potential to improve the micropropagation of *A. multiflora*, especially through adventitious shoot regeneration, axillary shoot multiplication, and rooting. CuO NPs showed a clear dose-dependent effect on all stages of *A. multiflora* micropropagation. Low concentrations of CuO NPs (5–10 mg/L) consistently enhanced adventitious shoot regeneration, axillary multiplication, and rooting, while higher concentrations were inhibitory, with complete suppression of morphogenesis at 40 mg/L CuO NPs. Leaves were the primary sites of antioxidant metabolite accumulation, although they were more sensitive to CuO NP-induced stress than microshoots. The dual role of CuO NPs as elicitors at low doses and inhibitors at high doses underscores their potential for optimizing tissue culture-based metabolite production. Overall, this work provides new insights into the interaction between nanomaterials and secondary metabolism during micropropagation and supports using controlled CuO NP supplementation to boost the production of valuable phytochemicals in medicinal plants.

## Figures and Tables

**Table 1:** Impact of CuO NP on adventitious shoot induction from leaf explants of *A. multiflora*.

CuO NP (mg/L)	Shoot Induction (%)	Number of Shoots/Explants	Shoot FW (g)
0	100 ± 0.0 a	23.7 ± 3.1 b	0.105 ± 0.01 b
5	100 ± 0.0 a	29.4 ± 4.1 a	0.107 ± 0.03 b
10	100 ± 0.0 a	20.4 ± 3.4 c	0.115 ± 0.02 b
20	100 ± 0.0 a	13.1 ± 2.2 d	0.151 ± 0.04 a
40	8.3 ± 2.5 b	1.1 ± 0.3 e	0.013 ± 0.003 c

Means ± SD within a column followed by the same lowercase letters (a–e) are not significantly different using DMRT at *p* ≤ 0.05.

**Table 2:** Impact of CuO NP on axillary shoot multiplication from leaf explants of *A. multiflora*.

CuO NPs (mg/L)	Shoot Induction (%)	Number of Shoots/Explants	Shoot FW (g)
0	100 ± 0.0 a	17.3 ± 2.4 b	0.211 ± 0.04 b
5	100 ± 0.0 a	22.4 ± 3.2 a	0.247 ± 0.03 ab
10	100 ± 0.0 a	15.9 ± 1.6 b	0.269 ± 0.02 a
20	100 ± 0.0 a	9.6 ± 2.8 c	0.247 ± 0.06 ab
40	71.1 ± 15.4 b	1.0 ± 0.0 d	0.106 ± 0.03 c

Means ± SD within a column followed by the same lowercase letters (a–d) are not significantly different using DMRT at *p* ≤ 0.05.

**Table 3:** Impact of CuO NP on rooting of *A. multiflora*.

CuO NPs (mg/L)	Root Induction (%)	Number of Roots/Shoots	Plantlet FW (mg)
0	100 ± 0.0 a	5.7 ± 0.9 b	1.71 ± 0.29 b
5	100 ± 0.0 a	10.2 ± 2.0 a	2.26 ± 0.24 a
10	100 ± 0.0 a	11.1 ± 3.6 a	1.35 ± 0.23 c
20	80.6 ± 8.1 b	4.8 ± 1.6 b	0.52 ± 0.07 d
40	0.0 ± 0.0 c	0.0 ± 0.0 c	0.0 ± 0.0 e

Means ± SD within a column followed by the same lowercase letters (a–e) are not significantly different using DMRT at *p* ≤ 0.05.

**Table 4:** Effect of various concentrations of CuO NPs on α-tocopherol and carotenoid contents (µg/g FW) in *A. multiflora*.

Organ	CuO NPs (mg/L)	α-Tocopherol (Retention Time (RT) 16.996 min)	(All-*E*)-Violaxanthin (RT 14.116 min)	9-*Z*-Neoxanthin (RT 15.059 min)	(All-*E*)-Lutein (RT 19.398 min)	(All-*E*)-β-Carotene (RT 31.771 min)	Total Carotenoids
Microshoot	0	26.5 ± 0.92 d	15.8 ± 0.8 e	12.1 ± 0.2 g	77.3 ± 2.5 f	25.5 ± 0.6 e	130.8 ± 3.0 f
	5	29.4 ± 1.4 d	17.6 ± 1.0 de	15.5 ± 1.0 ef	78.9 ± 1.3 f	23.7 ± 2.0 e	135.8 ± 5.4 ef
	10	28.6 ± 1.0 d	17.5 ± 1.3 de	13.8 ± 0.4 fg	80.7 ± 1.7 f	28.0 ± 1.7 e	140.0 ± 4.8 ef
	20	26.7 ± 0.5 d	21.1 ± 1.9 d	17.3 ± 1.2 e	90.6 ± 7.3 e	24.1 ± 2.3 e	153.1 ± 12.6 e
	40	-	-	-	-	-	-
Leaf	0	41.2 ± 2.6 c	53.5 ± 4.6 a	49.5 ± 0.8 b	220.9 ± 5.4 b	93.0 ± 5.0 b	417.0 ± 6.1 b
	5	50.3 ± 1.8 a	51.6 ± 1.2 a	54.2 ± 0.6 a	238.7 ± 2.0 a	102.6 ± 1.5 a	447.1 ± 2.1 a
	10	45.7 ± 0.7 b	39.2 ± 0.5 b	44.8 ± 1.2 c	210.1 ± 8.3 c	84.8 ± 11.5 c	378.9 ± 21.3 c
	20	44.6 ± 2.7 b	26.9 ± 2.3 c	27.3 ± 2.9 d	132.9 ± 7.7 d	39.4 ± 0.9 f	226.5 ± 13.8 d
	40	-	-	-	-	-	-
F-test	Org	<0.0001	<0.0001	<0.0001	<0.0001	<0.0001	<0.0001
	Con	<0.0001	<0.0001	<0.0001	<0.0001	<0.0001	<0.0001
	Org*Con	0.0336	<0.0001	<0.0001	<0.0001	<0.0001	<0.0001

Means ± SD within a column followed by the same lowercase letters (a–g) are not significantly different using DMRT at *p* ≤ 0.05.

**Table 5:** Effect of various concentrations of CuO NPs on sterols content (µg/g FW) in *A. multiflora*.

Organ	CuO NPs (mg/L)	22-Dehydroclerosterol	Clerosterol	Total Phytosterol
Microshoot	0	71.2 ± 0.3 f	168.3 ± 2.7 d	239.6 ± 2.4 e
	5	86.8 ± 4.5 d	214.4 ± 3.5 a	301.2 ± 8.0 b
	10	78.3 ± 1.7 e	201.3 ± 8.8 b	279.6 ± 7.2 c
	20	73.3 ± 2.7 ef	189.6 ± 3.9 c	262.9 ± 5.9 d
	40	-	-	-
Leaf	0	105.5 ± 4.8 ab	177.6 ± 6.5 cd	283.1 ± 11.3 c
	5	108.5 ± 3.6 a	210.5 ± 7.7 ab	319.1 ± 10.9 a
	10	101.4 ± 5.6 bc	181.6 ± 11.3 c	283.0 ± 15.8 c
	20	99.4 ± 0.9 c	168.6 ± 2.6 d	268.0 ± 3.5 cd
	40	-	-	-
F-test	Org	<0.0001	0.0049	0.0002
	Con	0.0001	<0.0001	<0.0001
	Org*Con	0.0214	0.0030	0.0053

Means ± SD within a column followed by the same lowercase letters (a–f) are not significantly different using DMRT at *p* ≤ 0.05.

**Table 6:** Effect of organ and CuO NPs treatment on fatty acids content (µg/g FW) in *A. multiflora*.

Organ	CuO NPs (mg/L)	Palmitic (RT 20.931 min)	Stearic (RT 24.562 min)	Oleic (RT 25.647 min)	Linoleic (RT 27.278 min)	Arachidic (RT 27.988 min)	α-Linolenic (RT 29.125 min)	Total SFAs	Total PUFAs	n-6/n-3 PUFAs
Microshoot	0	21.7 ± 0.77 a	3.8 ± 0.03 a	8.2 ± 0.31 d	25.7 ± 0.52 b	1.2 ± 0.08 a	39.5 ± 0.26 cd	26.6 ± 0.69 a	65.2 ± 0.39 c	0.65 ± 0.01 a
	5	20.0 ± 0.80 b	3.8 ± 0.31 a	10.7 ± 0.36 b	25.6 ± 2.27 b	1.1 ± 0.13 a	38.8 ± 3.14 d	24.8 ± 1.22 ab	64.4 ± 0.94 c	0.66 ± 0.11 a
	10	19.5 ± 0.33 b	3.7 ± 0.11 a	10.2 ± 0.53 bc	25.9 ± 0.21 b	1.1 ± 0.05 a	39.7 ± 1.21 cd	24.2 ± 0.48 b	65.7 ± 1.01 c	0.65 ± 0.03 a
	20	19.0 ± 0.24 b	3.8 ± 0.04 a	7.1 ± 0.42 e	28.3 ± 0.57 a	1.1 ± 0.03 a	40.6 ± 0.11 cd	23.9 ± 0.24 b	69.0 ± 0.64 b	0.69 ± 0.02 a
	40	-	-	-	-	-	-	-	-	-
Leaf	0	11.2 ± 0.28 c	1.8 ± 0.07 d	6.2 ± 0.17 f	18.7 ± 0.30 de	0.3 ± 0.04 d	61.6 ± 0.56 a	13.2 ± 0.31 d	80.3 ± 059 a	0.30 ± 0.01 d
	5	18.4 ± 0.24 b	2.7 ± 0.11 c	8.2 ± 0.07 d	19.8 ± 0.10 d	0.4 ± 0.01 c	50.4 ± 0.24 b	21.5 ± 0.18 c	70.2 ± 0.15 b	0.39 ± 0.01 c
	10	18.5 ± 0.88 b	3.1 ± 0.05 b	9.8 ± 0.24 c	17.0 ± 0.44 e	0.4 ± 0.05 c	51.0 ± 0.18 b	22.0 ± 0.87 c	68.0 ± 0.62 b	0.33 ± 0.01 cd
	20	18.8 ± 1.98 b	3.9 ± 0.35 a	13.6 ± 0.87 a	21.7 ± 1.2 c	0.6 ± 0.02 b	41.7 ± 1.96 c	23.3 ± 2.36 bc	63.4 ± 3.21 c	0.52 ± 0.01 b
	40	-	-	-	-	-	-	-	-	-
F-test	Org	<0.0001	<0.0001	0.0394	<0.0001	<0.0001	<0.0001	<0.0001	<0.0001	<0.0001
	Con	0.0002	<0.0001	<0.0001	<0.0001	0.0068	<0.0001	<0.0001	<0.0001	0.0002
	Org*Con	<0.0001	<0.0001	<0.0001	0.0768	0.0003	<0.0001	<0.0001	<0.0001	0.0125

Means ± SD within a column followed by the same lowercase letters (a–f) are not significantly different using DMRT at *p* ≤ 0.05. SFA: Saturated fatty acids, PUFA: Polyunsaturated fatty acids, n-6/n-3: Omega-6/omega-3.

## Data Availability

The original contributions presented in this study are included in the article/Supplementary Materials. Further inquiries can be directed to the corresponding author.

## Supplementary Materials

The following supporting information can be downloaded at: https://www.mdpi.com/article/10.3390/plants14243807/s1, Figure S1: Outline of the methodology employed for the extraction of crude lipids; Figure S2: Outline of the methodology employed for hydrolysis and the preparation of fatty acid methyl esters (FAMEs); Figure S3: Outline of the methodology employed for the hydrolysis and silylation of sterols for GC-MS analysis; Table S1: Liquid Chromatography (LC)–Mass Spectrometry (MS) parameters used for the analysis of carotenoids and tocols; Table S2: Gas Chromatography (GC)–Flame Ionization Detector (FID) Parameters used for the analysis of Fatty acid methyl esters (FAMEs); Table S3: Gas Chromatography (GC)–Mass Spectrometry (MS) parameters used for the quantitative analysis of sterols.
