# Supplementary material for: Impact of Copper Oxide Nanoparticles on Adventitious Shoot Regeneration, Axillary Shoot Multiplication, Rooting, and Bioactive Compounds in Ajuga multiflora Bunge
Source: Plants (Basel). 2025 Dec 13;14(24):3807. doi: 10.3390/plants14243807 (PMC12736757; doi:10.3390/plants14243807)
Supplement: Supplementary file 1 [file plants-14-03807-s001.zip › plants-4028398-supplementary.pdf]

Article

Impact of Copper Oxide Nanoparticles on Adventitious Shoot Regeneration, Axillary Shoot Multiplication, Rooting, and Bioactive Compounds in *Ajuga multiflora* Bunge

Iyyakkannu Sivanesan [1], Shuchi Upadhyay [2], Young-Soo Keum [3], Se Chul Chun [1] and Ramesh Kumar Saini [2,*]

[1] Department of Environmental Health Science, Human and Eco Care Center, Konkuk University, 1, Hwayang-dong, Gwangjin-gu, Seoul 05029, Republic of Korea; siva74@konkuk.ac.kr (I.S.); scchun@konkuk.ac.kr (S.C.C.)

[2] School of Health Sciences and Technology, UPES, Dehradun 248007, Uttarakhand, India; shuchi.upadhyay@ddn.upes.ac.in

[3] Department of Crop Science, Konkuk University, 1, Hwayang-dong, Gwangjin-gu, Seoul 05029, Republic of Korea; rational@konkuk.ac.kr

* Correspondence: rameshkumar.saini@ddn.upes.ac.in

Figure S1. Outline of the methodology employed for the extraction of crude lipids.

A 2.0 g sample of microshoots and leaves was placed into a 50.0 ml Falcon tube.

↓

The samples were homogenized with 25 ml of a solvent mixture composed of acetone, ethanol, and cyclohexane in a volumetric ratio of 1:1:2, containing 0.1% (w/v) butylated hydroxytoluene (BHT) as an antioxidant.

↓

Subsequently, the homogenates underwent bath sonication (JAC-2010; 300 W, 60 Hz) for 10 minutes, followed by ultra-shaking for 2 minutes using a Collomix Viba X.30 (Tinting Solutions B.V., Netherlands) to ensure efficient tissue disintegration and complete extraction.

↓

The resulting mixture was vacuum-filtered, and the residual pellets were subjected to a second extraction with 20 ml of the same solvent mixture.

↓

The combined filtrates, containing crude lipids (lipophilic compounds), were pooled and transferred to a 250 ml round-bottom flask, then vacuum-dried using a rotary evaporator at 35 °C.

↓

The crude lipids subsequently reconstituted in 4 ml of acetone containing 0.1% BHT and transferred to a 5 ml glass vial fitted with a Teflon-lined screw cap. These crude lipid extracts were then utilized for further analyses as described below:

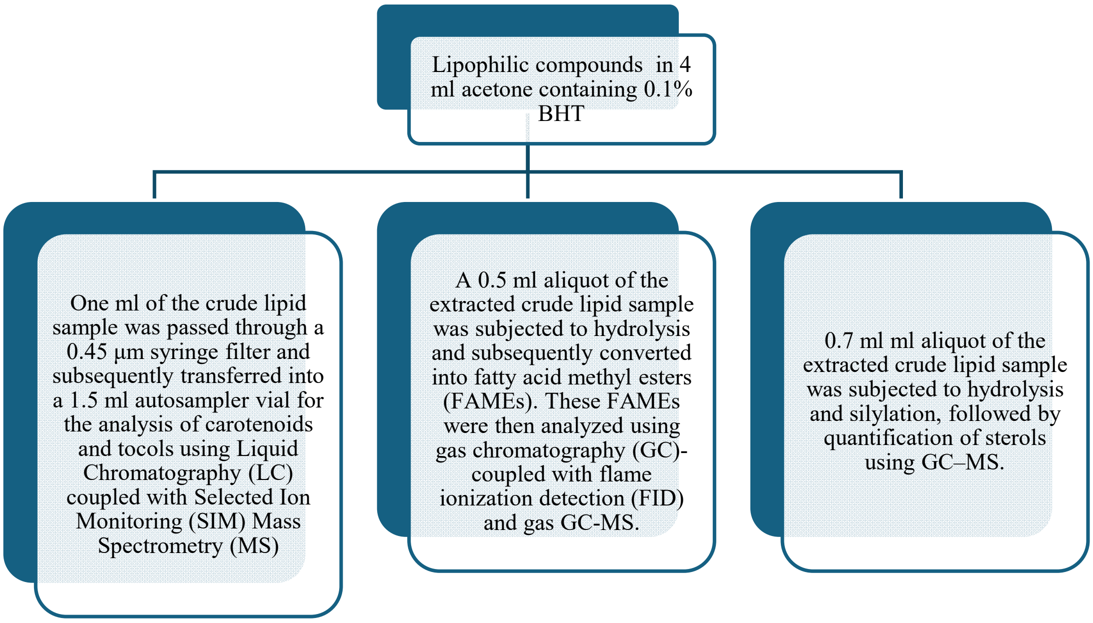

Figure S2. Outline of the methodology employed for hydrolysis and the preparation of fatty acid methyl esters (FAMEs).

A 0.5 mL aliquot of the extracted crude lipid sample was transferred into a 5.0 ml glass vial equipped with a PTFE-faced, rubber-lined cap.

↓

The solvent was subsequently removed by a nitrogen evaporator.

↓

Following evaporation, 1 ml of 0.5 M methanolic potassium hydroxide (KOH) was added, and the mixture was incubated at 85 °C for 30 minutes in a heat block to facilitate hydrolysis of crude lipids.

↓

After cooling, 1 mL of a 14% boron trifluoride (BF_3) solution in methanol was introduced, and the sample was incubated for an additional 20 minutes at 60 °C in the heat block.

↓

Upon cooling, 2 ml of cyclohexane (optionally, hexane can be added) and 1 ml of 1 % sodium chloride (NaCl) solution were added, the tube was thoroughly vortexed and allowed to separate into the upper organic (cyclohexane) phase and lower aqueous phase.

↓

The upper cyclohexane layer (1 ml) was then carefully recovered, filtered through a 0.45 µm PTFE syringe filter containing a small amount of anhydrous sodium sulfate to remove residual moisture, and transferred into a 1.5 ml autosampler vial for subsequent analysis by GC-FID and GC-MS.

Figure S3. Outline of the methodology employed for the hydrolysis and silylation of sterols for GC-MS analysis.

A 0.7 ml aliquot of the extracted crude lipid sample was transferred into a 5.0 ml glass vial equipped with a Teflon-lined screw cap, to which 10 µg of the internal standard for phytosterols, 5β-cholestan-3α-ol, was added.

↓

The sample was then evaporated to dryness using a nitrogen evaporator.

↓

Subsequently, 2 ml of 0.5 M potassium hydroxide (KOH; in methanol) was added, and the mixture was incubated in a water bath at 85 °C for 15 minutes to facilitate hydrolysis of crude lipids.

↓

Following hydrolysis, the samples were immediately cooled on ice and subjected to liquid-liquid partitioning with 2 ml of cyclohexane and 1 ml of 1% sodium chloride (NaCl).

↓

From the upper cyclohexane phase, which contained the phytosterols, 1 ml was carefully collected and transferred into a new 5.0 ml Teflon-lined glass tube, then dried a nitrogen evaporator.

↓

For the silylation of phytosterols, 1 ml of pyridine and 30 µl of N,O-bis(trimethylsilyl)trifluoroacetamide (BSTFA) containing 1% trimethylchlorosilane (TMCS) were added, and the mixture was incubated at 65 °C for 60 minutes.

↓

After incubation, the reaction mixture was cooled on ice, filtered through a 0.45 µm polytetrafluoroethylene (PTFE) syringe filter, and transferred into a 1.5 ml autosampler vial for subsequent GC-MS analysis.

Table S1. Liquid Chromatography (LC)- Mass Spectrometry (MS) parameters used for the analysis of carotenoids and tocols.

**HPLC: Nexera 40 series Ultra-high Performance Liquid Chromatograph (UHPLC; Shimadzu, Tokyo, Japan)**	
**Column**	YMC carotenoid (C_{30}) column (3 µm,150 mm, 4.6 mm; YMC, Wilmington, NC)
**Mobile phase**	**Solvent A**- methanol/water (95/5; v/v) containing 5 mM ammonium formate (NH_4HCO_2; LC-MS grade)<br>**Solvent** B- tert-butyl methyl ether (tBME)/methanol/water (90/7/3, v/v/v), containing 5 mM ammonium formate
**Injection volume (µl)**	2
**Flow rate (ml/min)**	0.5
**Gradient program**	100 % A (0.0 min) –0 % A (45.0 min); 5-min post-run (100 % A)
**Column oven temperature (°C)**	20
**MS: LCMS-9030 quadrupole time-of-flight (Q-TOF) spectrometer (Shimadzu, Tokyo, Japan)**	
**Ionization**	Atmospheric-pressure chemical ionization (APCI) in positive mode
**MS program**	(0 min to 8.0 min)- diverter valve to drain; (8.01 to 45.0 min)- diverter valve to MS
**Interface temperature (°C)**	400
**Nebulizing gas flow (L/min)**	3
**DL temperature (°C)**	300
**Drying gas flow (L/min)**	10
**Corona needle voltage (kv)**	4.0
**Heat block Temperature (°C)**	300
**Screening mode**	Single Ion Monitoring (SIM)
**Data acquisition (sampling)**	1.85625 Hz
**Q1 resolution (ppm)**	± 20

Table S2. Gas Chromatography (GC)-Flame Ionization Detector (FID) Parameters used for the analysis of Fatty acid methyl esters (FAMEs).

**Parameters**	**Value**		
**Injection port temperature (°C)**	250		
**Injection mode**	Split (5:1)		
**Split flow (ml/min)**	10		
**Injection volume (µl)**	1		
**Inlet total flow (ml/min)**	15		
**Inlet pressure (psi)**	54.901		
**Carrier Gas Flow ml/min (Nitrogen)**	2		
**Septum purge flow (ml/min)**	3		
**Detector airflow (ml/min)**	400		
**Detector H_2 flow (ml/min)**	30		
**Detector makeup flow ml/min (Nitrogen)**	25		
**Column**	Supelco SP-2560 capillary (100 m, 0.25mm ID, 0.20 µm film thickness; Merck KGaA, Darmstadt, Germany).		
**Column over temperature**	Rate (°C/min)	Final temperature (°C)	Hold time (min)
	-	140.0	1
	5.0	240.0	15
**Detector**	Flame ionization detector (FID)		
**Run time (min)**	45		
**Post run time (min)**	5		
**Detector temperature (°C)**	260		

Table S3. Gas Chromatography (GC)-Mass Spectrometry (MS) parameters used for the quantitative analysis of sterols.

**Gas chromatograph**			
**Injection Temperature (°C)**	**260**		
**Column over temperature (°C)**	150		
**Injection mode**	split		
**Flow control mode**	Liner velocity		
**Pressure (kPa)**	86.5		
**Carrier gas**	Helium		
**Total Flow (ml/min)**	8.6		
**Column Flow (ml/min)**	0.93		
**Purge flow (ml/min)**	3.0		
**Liner velocity (cm/sec)**	36.7		
**Column**	DB-5ms (30 m, 0.25 mm ID, 0.25 μm film thickness; Agilent Technologies Canada, Inc.)		
**Column over temperature**	Rate (°C/min)	Final temperature (°C)	Hold time (min)
	-	150.0	1
	20.0	300.0	30
**Total program time (min)**	38.5		
**Mass spectrometer**			
**Ion source temperature (°C)**	260		
**Start time (min)**	6		
**Interface temperature (°C)**	280		
**Solvent cut time (min)**	3		
**Event time (sec)**	30		
**End time (min)**	38		

**Acquiring mode**	Scan
**Start m/z**	50
**Scan speed**	2500
**End m/z**	650
